# Scaling‐up the Bioconversion of Lignin to 2,4‐Pyridinedicarboxylic Acid With Engineered *Pseudomonas putida* for Bio‐Based Plastics Production

**DOI:** 10.1002/bit.70020

**Published:** 2025-07-14

**Authors:** Jan Seeger, Susanne Müller, Helena Gómez‐Álvarez, Goran M. M. Rashid, Timothy D. H. Bugg, Eduardo Díaz, Ralf Takors

**Affiliations:** ^1^ Institute of Biochemical Engineering University of Stuttgart Stuttgart Germany; ^2^ Margarita Salas Center for Biological Research Spanish National Research Council Madrid Spain; ^3^ Department of Chemistry University of Warwick Coventry UK

**Keywords:** 2,4‐pyridinedicarboxylic acid, bio‐based plastics, lignin valorization, mixed culture, *Pseudomonas putida*, *Rhodococcus jostii*

## Abstract

2,4‐pyridinedicarboxylic acid (PDCA) is a promising bio‐based compound to substitute petroleum‐derived terephthalic acid in plastics. It is produced through the microbial conversion of lignin substrates with engineered microorganisms like *Pseudomonas putida*. To this point, an efficient bioproduction process for PDCA has not yet been established. In this study, we optimized PDCA production with engineered *P. putida ligAB* and demonstrated bioproduction at up to 30 L scale. PDCA was produced with a volumetric productivity of 390 mg/L/h from the precursor protocatechuate and the product titer was doubled compared to previously reported work. Lignin feedstock and pretreatment combinations were screened to access lignin as substrate for PDCA production. Sodium hydroxide lignin with alkali + heat pretreatment yielded 33 mg/L PDCA at a production rate of 0.1 mg/g/h. Low PDCA production rates could be overcome by developing a bacterial mixed culture by adding the engineered strain *Rhodococcus jostii ΔpcaHG* that supplies PCA from lignin degradation. The mixed culture increased PDCA productivity of *P. putida ligAB* by factor 19 (1.9 mg/g_*P. putida*_/h).

**Abbreviations**
HPLC: high performance liquid chromatography
PCA: protocatechuic acid
PDCA: 2,4‐pyridinedicarboxylic acid
*P. putida*
: *Pseudomonas putida* KT2440
*R. jostii*
: *Rhodococcus jostii* RHA1
SH: sodium hydroxide

## 1 Introduction

Lignin is a broadly available, renewable aromatic carbon source, which has rarely been applied in biomanufacturing to date. As part of lignocellulose, the main component of the plant cell wall, lignin is synthesized from three different phenylpropanoid units: p‐coumaryl alcohol (H), coniferyl alcohol (G), and sinapyl alcohol (S) through radical polymerization (Boerjan et al. 2003). About 300 billion tons of lignin are globally available with a rising offer of 20 billion tons annually (Becker and Wittmann 2019). Right now, the cellulose fraction of lignocellulose is primarily utilized as starting material for pulp and paper manufacturing and as feedstock for bioethanol production (Brown and Brown 2013; Pauly and Keegstra 2008). Among the different processes for lignocellulose disintegration Kraft pulping is dominating accounting for about 130 million tons of produced lignin annually (Rinaldi et al. 2016). The remaining lignin fraction is mainly considered for combustion (Becker and Wittmann 2019).

Biotechnological lignin valorization is a promising alternative to make use of the largely available resource with a high content of aromatic carbon. Hereby, the natural potential of (micro‐) organisms to degrade lignin is exploited to synthesize industrially relevant products such as muconic acid, adipic acid, PHA or lipids (Kumar et al. 2019; Li et al. 2019; Sonoki et al. 2018; Vardon et al. 2015). This opens the door for the sustainable biomanufacturing of bio‐plastics from the above‐mentioned monomers. So far, fully bio‐based plastics that can compete with production costs and material properties of fossil‐oil‐based plastics are still rare (Nguyen et al. 2018).

**Figure 1:**
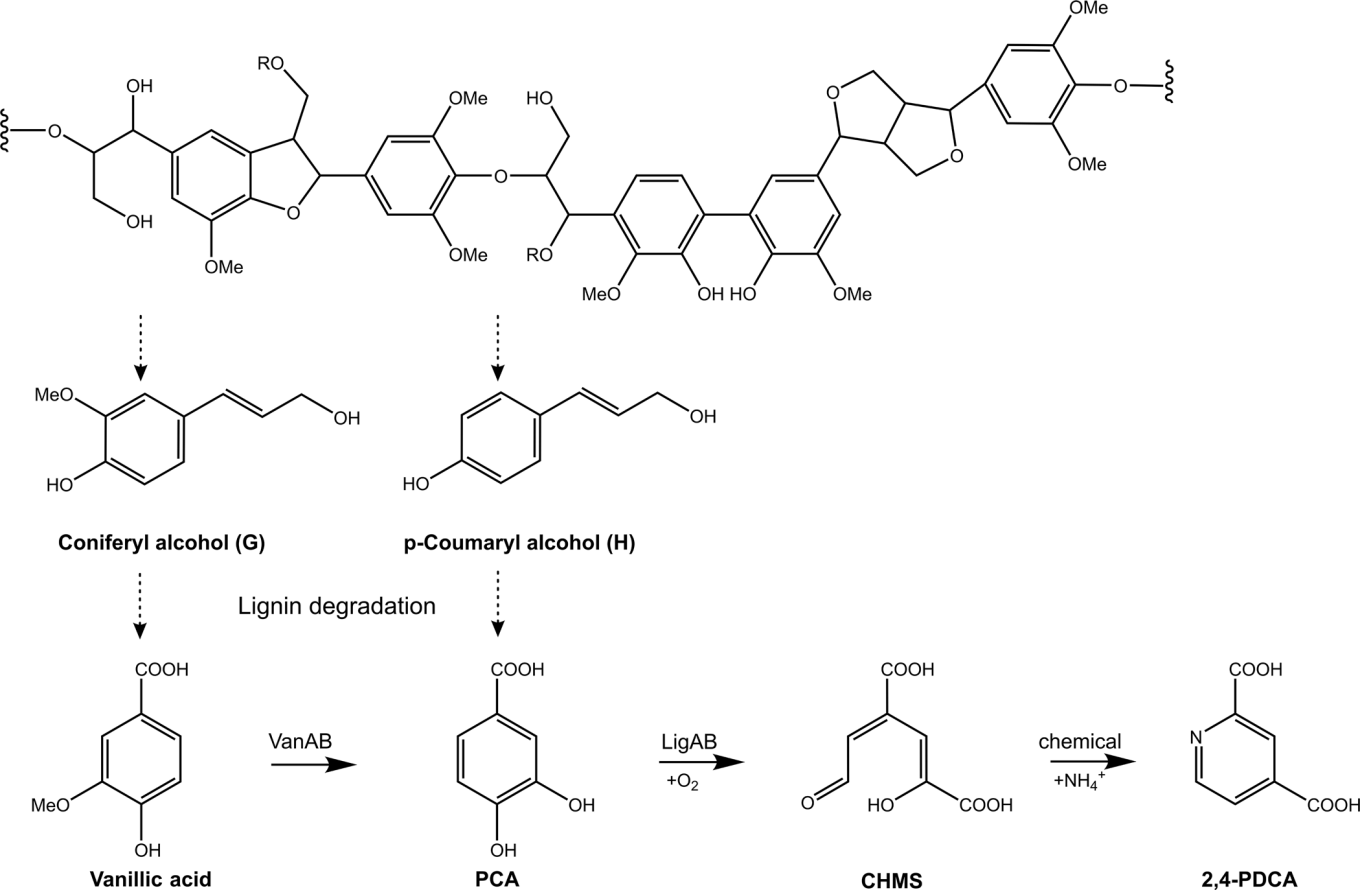
Metabolic scheme from lignin to 2,4‐pyridinedicarboxylic acid (PDCA). Protocatechuic acid (PCA) is one major metabolite generated in aromatic funnelling of lignin‐derived G‐ and H‐ monolignols. PCA metabolism in *P. putida* is redirected towards formation of PDCA via intermediate 4‐carboxy‐2‐hydroxymuconate‐semialdehyde (CHMS) by replacing the *pcaG* gene (encoding the intradiol protocatechuate‐3,4‐dioxygenase) with the heterologous *ligAB* genes (encoding the extradiol protocatechuate‐4,5‐dioxygenase). The endogenous *vanAB* genes encode the vanillate monooxygenase. Dotted arrows indicate several reaction steps. Scheme modified from Ahmad et al. 2010 and Spence et al. 2021.

To fill the gap, Mycroft et al. 2015 engineered the soil bacterium *Rhodococcus jostii* RHA1 targeting the production of pyridinedicarboxylic acids (PDCA) from lignin. PDCA are analogs to terephthalic acid, one major dicarboxylic acid additive for polymer production (e.g. polyethyleneterephthalate PET or polybutyrate adipate terephthalate PBAT) with an annual production of over 80 million tons worldwide (statista 2023). Hence, polyesters from PDCA offer the potential to substitute fossil‐oil‐based plastics (Pellis et al. 2019). By integrating the heterologous genes of protocatechuate‐4,5‐dioxygenase (LigAB) from *Sphingobium lignivorans* SYK‐6 into the soil bacterium *R. jostii* the conversion of protocatechuate (PCA) was re‐routed towards 2,4‐pyridinedicarboxylic acid (Figure 1, Barry and Taylor 2013; Mycroft et al. 2015; Spence et al. 2021). PCA is one major monoaromatic intermediate from bacterial degradation of G‐ and H‐lignin units (Chen and Wan 2017). The LigAB enzyme catalyzes the extradiol 4,5‐ring cleavage of protocatechuate to form 4‐carboxy‐2‐hydroxymuconate semialdehyde (CHMS), which chemically reacts with ammonium leading to spontaneous cyclisation to form 2,4‐PDCA (Figure 1) (Barry and Taylor 2013; Mycroft et al. 2015; Shindo et al. 2004).

Recently, Gómez‐Álvarez et al. 2022 implemented the production of 2,4‐PDCA in *Pseudomonas putida* KT2440, which is a promising industrial host for lignin valorization (Ankenbauer et al. 2020; Johnson et al. 2019; Kumar et al. 2021). A resting‐cell process using the recombinant strain *P. putida ligAB* (harboring a substitution of the chromosomal *pcaG* gene with the *ligAB* genes) showed more than 15 times higher productivity for the conversion of lignin‐derived monoaromatics to PDCA than the previously engineered *R. jostii* expressing *ligAB* genes (Gómez‐Álvarez et al. 2022). However, side product formation occurred that limited the PDCA molar conversion yield to 43%. A fraction of the CHMS reacted to form a cyclic hemiacetal that accumulated. Maximum PDCA titer was 0.9 g/L from 1.5 g/L PCA and 40 mg/L from 15 g/L sodium hydroxide (SH) lignin. Although efforts were done to prevent the formation of the hemiacetal form, e.g. increasing pH (up to 9) to promote the open form of CHMS and thus its cyclization to PDCA in the presence of ammonium, no increase of the conversion yield to PDCA was observed (Gómez‐Álvarez et al. 2022).

An industrially relevant PDCA production process is still missing. Given that monomers for large‐scale plastic production are commodities, any production process must operate with maximum conversion yield and productivities for competing with the established fossil‐based routes. Hence, this study aims to optimize and transfer PDCA production with *P. putida ligAB* from shake flask into a bioreactor scale ensuring high yield and productivity. First, a production protocol was developed to increase the PDCA molar conversion yield from PCA. Thereon, the bioprocess was engineered to produce PDCA in up to 30 L reactor scale with resting cells. Lignin is a challenging substrate for bioconversion due to its low solubility and recalcitrant structure. To make it accessible for PDCA production, different lignin substrates and pretreatments were screened to reveal the best combination. PDCA production from lignin was achieved but realizing high productivities was still challenging. Interestingly, a mixed culture combining the PDCA‐producer *P. putida ligAB* with the lignin‐degrading *R. jostii ΔpcaHG* significantly increased the PDCA production from lignin.

## 2 Materials and Methods

### 2.1 Bacterial Strains and Cultivation

*Pseudomonas putida KT2440 ΔpcaG:ligAB* (*P. putida ligAB*) was engineered by Gómez‐Álvarez et al. 2022. *P. putida ligAB* bears a chromosomal replacement of the *pcaG* gene (protocatechuate‐3,4‐dioxygenase alpha chain) by *ligAB* (protocatechuate‐4,5‐dioxygenase) from *Sphingobium lignivorans SYK‐6* under control of the inducible promotor *Ptac*. The genetic engineering enables the strain to accumulate 2,4‐PDCA from protocatechuate (PCA) conversion. Thereby, the metabolization of PCA is disabled by knockout of *pcaG*, which represents the first enzyme in the β‐ketoadipate pathway for PCA metabolization. *Rhodococcus jostii RHA1 ΔpcaHG* (*R. jostii ΔpcaHG*) was engineered by Spence et al. 2021. *R. jostii ΔpcaHG* harbors the chromosomal knock‐out of the *pcaHG* (protocatechuate‐3,4‐dioxygenase alpha and beta chain) genes. This allows the strain to accumulate PCA from lignin conversion instead of metabolizing it via β‐ketoadipate pathway. Other than reported in Spence et al. 2021, the strain does not harbor the *ligAB* genes for 2,4‐PDCA production in this study. Both strains were cultivated at 30°C in M9 minimal medium as described in Spence et al. 2021 with 5–10 g/L glucose. All chemicals for cultivations were obtained from Carl Roth, Germany unless stated otherwise. A correlation factor of 0.3 g/L (data not shown) was used to calculate the biomass concentration from the OD 600 nm measurements.

### 2.2 PDCA Production From PCA With *P. putida ligAB*

#### 2.2.1 Biotransformation in Shake Flask

The protocol for producing PDCA from PCA in resting cells was adapted from Gómez‐Álvarez et al. 2022. Seed culture *of P. putida ligAB* grown in M9 medium was added to 200 mL main culture in M9 medium. IPTG was added for different durations to induce the expression of *ligAB*. For 3 h induction duration, 0.5 mM IPTG were added after 3 h and incubated for further 3 h as described in (Gómez‐Álvarez et al. 2022). For 6 and 15 h induction duration, 1 mM IPTG was added from the beginning of the main culture and incubated for the indicated time. This approach allowed harvesting the cells during growth phase while also extending the duration of induction. Cells were harvested by centrifugation and resuspended in 10 mL reaction buffer to reach a final biomass concentration of 10 g/L in the reaction. The reaction buffer contained 50 mM sodium phosphate buffer at pH 7.5 with 0.1 M NH_4_Cl and 0.8 g/L PCA (Thermo Scientific, USA) and no carbon source that could support cell growth to obtain resting cells. The biotransformation was performed at 30°C on a rotary shaker in a 50 mL baffled flask.

The molar conversion yield is defined as the molar percentage of PDCA produced from PCA. The volumetric productivity describes the mass of PDCA produced per litre and hour. The biomass‐specific productivity was calculated from the slope of PCA or PDCA concentration over time (time interval indicated in brackets) per biomass concentration in the reaction.

Values were compared by one‐way ANOVA with the 3 h results as reference and a significance level of *α* = 5%.

#### 2.2.2 Cultivation and Biotransformation in Bioreactor

For cultivation in bioreactors, an enriched M9 medium with 10 g/L glucose was used with following composition 8 g/L Na_2_HPO_4_ x 2 H_2_O, 4 g/L KH_2_PO_4_, 1 g/L NaCl, 1.2 g/L NH_4_Cl, 0.1 g/L FeSO_4_ x 7 H_2_O, 0.5 g/L Na_3_‐Citrate x 2 H_2_O, 0.79 g/L MgSO_4_ x 7 H_2_O, 0.9 g/L CaCl_2_ x 2 H_2_O, and 1.2 mL trace element solution. The trace element solution was prepared from 0.5 g/L FeSO_4_ x 7 H_2_O, 0.4 g/L ZnSO_4_ x 7 H_2_O, 0.02 g/L MnSO_4_ x H_2_O, 0.015 g/L H_3_BO_3_, 0.01 g/L NiCl_2_ x 6 H_2_O, 0.25 g/L ethylenediaminetetraacetic acid (EDTA), 0.027 g/L CoCl_2_, 0.05 g/L CuCl_2_ x 2 H_2_O, 2 g/L NaMoO_4_ x 2 H_2_O, and 5.22 g/L Na‐EDTA x 2 H_2_O.

*P. putida ligAB* was cultivated in duplicates in 1.5 L glass bioreactors (Eppendorf, Germany) and in 30 L stainless steel bioreactor (Bioengineering, Switzerland) at 30°C. The pH value was controlled at pH 7.0 during the growth phase but was raised to 7.5 for the production phase with 25% NH_4_OH. Thereby, the pH of 7.5 corresponds to the pH optimum for LigAB (Barry and Taylor 2013). The dissolved oxygen was maintained above 30% by controlling the stirrer speed in the range from 200 to 1000 rpm. The reactors were inoculated to a starting OD_600 nm_ of ~0.1 in an initial volume of 0.5 L/8 L with cells from seed culture. After initial batch phase, the exponential (*µ* = 0.1 1/h) glucose feed was started comprised of 400 g/L glucose dissolved in enriched M9 medium. IPTG was added from the start and supplemented with the glucose feed at final concentrations of 0.5 mM (30 L bioreactor) or 1.0 mM (1.5 L bioreactor). After growth phase, PDCA production phase was initiated by stopping the glucose feed and starting the PCA feed at a constant rate of 0.8 g/L/h from a concentrated PCA stock in water at pH 7.5. The start of PCA feed is defined as time point 0 h. At that time, glucose was immediately depleted—as reflected in the dissolved oxygen peak—and was not further supplemented to maintain resting cells. The initial dried biomass and volume at start of PCA feed was 17 g/L in 0.5 L for 1.5 L bioreactors and 36 g/L in 11.0 L for 30 L reactor. The 1.5 L bioreactor volume was adjusted after growth phase to start PDCA production phase with 0.5 L total volume.

### 2.3 Lignin as Substrate

#### 2.3.1 Substrate and Pretreatment Screening

Sodium hydroxide (SH) lignin was originally obtained from Greencone Environs, India. Kraft lignin was purchased from Merck, Germany and wheat straw was provided by a local farmer. All lignin substrates were obtained as water free powder. Dried wheat straw was chopped to ~1 cm pieces. Lignin substrate was added to deionized water at a concentration of 20 g/L and processed following four different protocols: I. untreated control, II. autoclaving (120°C, 20 min), III. adapted from Zhao et al. 2021: setting pH to 13 for 1 day, adjusting pH to 7.4 before adding to the culture and IV. same as III. but with autoclaving at pH 13. pH was adjusted using 10 M NaOH and 5 M HCl. The experiment was performed with 4 g/L resting cells of *P. putida ligAB* or *R. jostii ΔpcaHG* in M9 medium with 10 g/L SH or Kraft lignin or wheat straw as sole carbon source from the 20 g/L lignin solutions at a final volume of 20 mL. Cells were obtained from seed culture in M9 medium with 5 g/L glucose and harvested by centrifugation. The biomass pellet was resuspended with the lignin medium and the cells were incubated for 5–7 days at 30°C, shaking. No IPTG was added at any time to leave the promotor of *ligAB* uninduced in *P. putida ligAB*. Still, small amounts of PDCA were produced. Therefore, the lumped amount of PDCA and PCA (Equation 1) was used to compare the lignin conversion.

(1) P(D)CA=PDCA+PCA[mg/L]

The maximum values from different pretreatments and substrate combinations were compared by two factorial ANOVA and subsequent Tukey post hoc test with a significance level of *α* = 5%.

#### 2.3.2 Lignin Conversion in Bioreactor

SH lignin (5.7 g/L final) was used as substrate for PDCA or PCA production in 1.5 L bioreactor with *P. putida ligAB* or *R. jostii ΔpcaHG*. Therefore, a similar 2‐stage process was performed as described in Section 2.2.2 but with an initial volume of 0.9 L. *P. putida ligAB* was cultivated at pH 7.0 and *R. jostii ΔpcaHG* at pH 7.4. After growth phase, PDCA production was started by adding lignin substrate. A 40 g/L SH lignin solution was prepared by alkali + heat pretreatment (Section 2.3.1). The time of lignin pulse was defined as 0 h. For lignin conversion, the pH was set to 7.5 for both strains. The reaction volume was 1 L with biomass concentrations of 13 g/L *P. putida ligAB* or 17 g/L *R. jostii ΔpcaHG*. The cultivation and lignin conversion was performed as duplicates. Antifoam agent (Struktol J647 A, Schill+Seilacher, Germany) was added to the culture after lignin addition to reduce foaming.

### 2.4 Mixed Culture

Biomass for mixed culture experiments was harvested from bioreactor cultivations after growth in the presence of 0.5 mM IPTG for *P. putida ligAB*. Cells of *P. putida ligAB* were mixed with M9 medium without glucose to reach final concentrations of 3 or 6 g/L for single culture. For mixed culture experiments, cells of *P. putida ligAB* were mixed with *R. jostii ΔpcaHG* to reach final biomass concentration of 15–16 g/L. Therefore, 3 or 6 g/L *P. putida ligAB* were added to 13 or 9 g/L *R. jostii ΔpcaHG* in 18 mL total volume. PDCA production was started by adding 6.7 g/L SH lignin from a 40 g/L alkali and heat pretreated lignin stock. The time of lignin addition was defined as 0 h. The biomass was incubated shaking at 130 rpm, 30°C and an initial pH of 7.5 in a shake flask.

### 2.5 High Performance Liquid Chromatography Analysis

PDCA and the precursor PCA were quantified by HPLC. The method was modified from Gómez‐Álvarez et al. 2022. A 500 µL sample from the culture suspension was mixed with 500 µL HPLC buffer: 0.25 M HCl in 50% methanol (VWR International, USA) to stop the reaction and acidify the sample. Samples were centrifuged at 20,000 g for 5 min to remove precipitates, biomass, and lignin. The supernatant was used for injecting 10 µL into an Agilent 1260 Infinity HPLC device (Agilent Technologies, USA) equipped with a ZORBAX Eclipse Plus C18 column (4.6 × 250 mm, 5 μm, Agilent Technologies, USA). The solvents were A 0.1% trifluoracetic acid in water and B 0.1% trifluoracetic acid in methanol. PDCA was detected at 280 nm and PCA was detected at 260 nm with a diode array detector. For samples from experiments without lignin in the reaction, the solvent gradient was 15‐50% B for 12.5 min, 50‐15% B for 1.5 min, 15% B for 6 min at a constant flow rate of 0.9 mL/min. For samples from lignin conversion experiments, the solvent gradient was 15‐50% B for 10 min, 50‐70% B for 10 min and 70‐15% B for 10 min at a constant flow rate of 0.5 mL/min. Authentic standards of PDCA (Merck, Germany) and PCA (Thermo Scientific, USA) were used to calibrate the peak areas and calculate concentrations.

## 3 Results and Discussion

### 3.1 PDCA Production From the Monoaromatic PCA in *P. putida ligAB*

#### 3.1.1 Improved PDCA Production From PCA by Enhanced Induction of *ligAB*

PCA is a central intermediate derived from lignin degradation of H‐ and G‐ lignin units. Gómez‐Álvarez et al. 2022 re‐routed PCA metabolism in *P. putida ligAB* towards PDCA production. The conversion of PCA to PDCA includes two reaction steps: the oxidative benzene ring cleavage of PCA to 4‐carboxy‐2‐hydroxymuconate‐semialdehyde (CHMS) catalyzed by protocatechuate‐4,5‐dioxygenase (LigAB) and the ring cyclization after chemical reaction with ammonium (Figure 1). Despite production of 0.9 g/L PDCA from PCA, the spontaneous formation of the hemiacetal form of CHMS limited the conversion yield to 43%. The authors reported that attempts to prevent hemiacetal formation and increase PDCA yield by altering the reaction pH or increasing the amount of ammonium in the reaction were not successful. Hemiacetal formation not only reduced the achievable PCA‐to‐PDCA conversion yield but also contributed to growth inhibition. Consequently, best performance of *P. putida ligAB* was achieved with resting cells (Gómez‐Álvarez et al. 2022). Accordingly, PDCA production experiments in this study were also performed with resting cells. Therefore, biomass was first grown and *ligAB* expression was induced with IPTG. Afterwards, the cells were harvested for biotransformation in reaction buffer with aromatic or lignin substrates but without any growth substrates.

**Figure 2:**
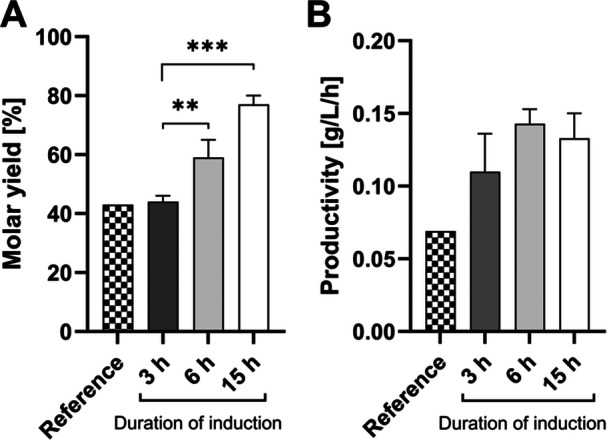
PDCA production from PCA with resting cells of *P. putida ligAB*. Cells were grown on glucose and incubated for 3, 6 or 15 h with the inducer IPTG before harvesting for biotransformation by centrifugation. 10 g/L biomass were resuspended in 50 mM phosphate buffer at pH 7.5 with 0.8 mg/L PCA as substrate for PDCA production. (A) The molar yield after 24 h and (B) volumetric productivity within the first 2 h of reaction were calculated. For reference, the values previously reported by Gómez‐Álvarez et al. 2022 were plotted (Reference). The data represents the mean of triplicates with standard deviation (error bars). Values were compared by one‐way ANOVA with *p* < 0.002 (**), and *p* < 0.001 (***).

The current study investigated the effect of enhanced induction of *ligAB* expression on the PDCA conversion with 10 g/L resting cells of *P. putida ligAB*. Purposefully, the duration of induction and inducer concentrations in the growth phase were evaluated. Interestingly, little PDCA production (62 ± 16 mg/L) was already observed even in uninduced cells. Increasing the inducer concentration above 0.5 mM led only to minor improvements (Supporting Information S1: Figure A.1A). However, extending the duration of induction in the growth phase from originally 3 h to overnight (15 h) significantly increased the molar conversion yield from 43% up to 77% (Figure 2). Maximum volumetric productivity was measured with 140 ± 10 mg/L/h for cells previously incubated for 6 h with inducer before harvesting for biotransformation. This corresponds to a biomass specific productivity of 14 ± 1 mg/g/h (0–2 h). However, other induction durations did not reveal statistical differences regarding the productivity.

Apparently, the extended induction duration during biomass growth enabled higher expression levels of the enzyme LigAB that catalyses the ring cleavage of PCA. As the productivity for PDCA did not improve proportionally with the duration of induction of *ligAB* it is anticipated that the downstream conversion from CHMS to PDCA or PCA uptake still limits productivity. Here, measurements of CHMS to PDCA conversion could help to identify the rate of CHMS conversion. However, CHMS is not commercially available. Regarding PCA uptake, future studies with cell extract containing overproduced LigAB enzyme could provide some hints on the existence of a potential PCA uptake bottleneck.

#### 3.1.2 Scale‐Up of PDCA Production From PCA in *P. Putida ligAB*

**Figure 3:**
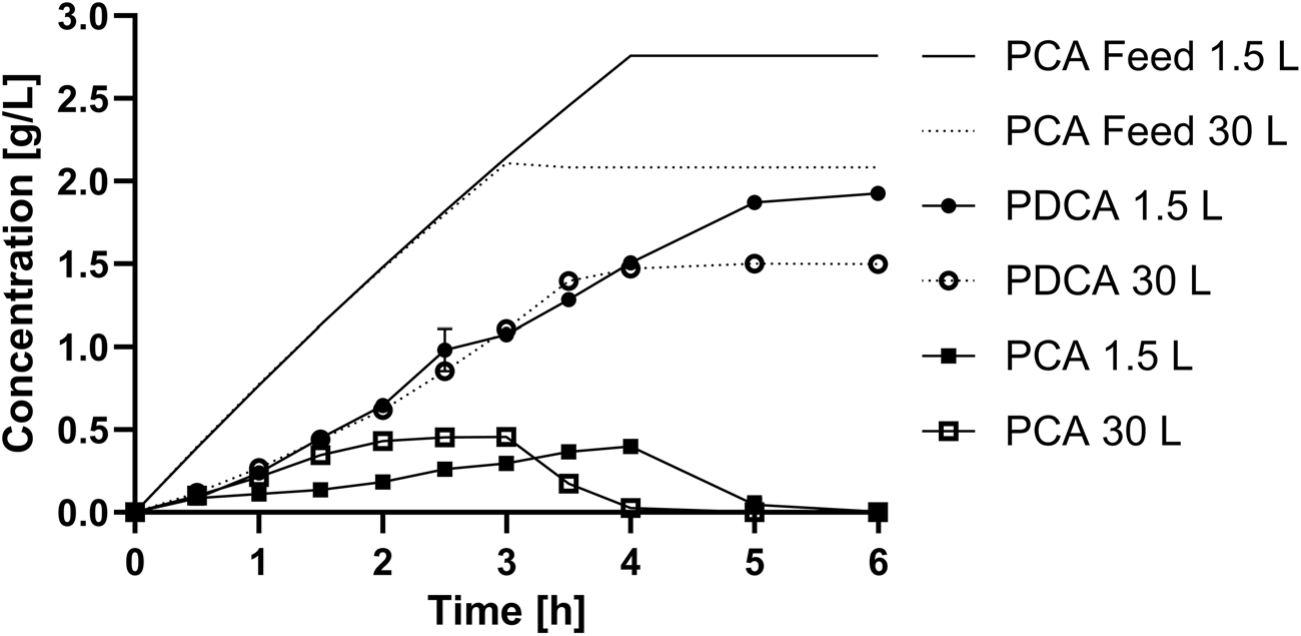
Bioreactor PDCA production from PCA in 1.5 and 30 L scale with *P. putida ligAB*. A two‐stage process was applied for bioreactor PDCA production. First, *P. putida ligAB* was grown on glucose in M9 medium in the presence of IPTG. Second, PDCA production started after depleting glucose and instead feeding PCA at a constant rate of 0.8 g/L/h (line) to resting cells of *P. putida ligAB*. The start of PCA feed is defined as *t* = 0 h. PDCA (dots) and PCA (squares) in the reactor were monitored. The experiment was performed in 1.5 L bioreactors in duplicates and in 30 L bioreactor. The data represents the mean of duplicates with variation (error bars) for 1.5 L bioreactor.

PDCA production was transferred from shake flask to a bioreactor scale. Therefore, a two‐stage process was applied: I. biomass production from glucose and II. glucose depletion and PDCA production from PCA as sole carbon substrate with resting cells. PCA was steadily fed with 0.8 g/L/h corresponding to the PCA uptake rate of 770 mg/L/h with 10 g/L biomass in shake flasks (Gómez‐Álvarez et al. 2022). The experiment was performed in 1.5 L bioreactors as well as in 30 L bioreactor (Figure 3). During PCA feeding, PDCA was constantly produced with a volumetric productivity of 394 ± 10 and 390 mg/L/h in the 1.5 and 30 L bioreactor, respectively. Small amounts of PCA accumulated during feeding to a maximum of 0.5 g/L and were converted to PDCA after stop of the PCA addition. The amount of biomass was monitored throughout the experiment with no notable changes of biomass during PDCA production phase. Overall, 1.9 ± 0.0 g/L PDCA were produced in 1.5 L bioreactor and 1.5 g/L in 30 L bioreactor. Both approaches showed similar kinetics which demonstrates the scalability. In 30 L reactor, the PCA feed was stopped earlier, since the feed was depleted. This resulted in a reduced final PDCA titer for 30 L compared to 1.5 L bioreactor. In total a conversion of 64 ± 1% (1.5 L bioreactor) and 61% (30 L bioreactor) of PCA to PDCA was achieved at the end of the process.

The current approach doubled the benchmarking PDCA titers (Gómez‐Álvarez et al. 2022) and further demonstrated volumetric scale‐up to 30 L. As there was no decline in performance during the 6 h production phase, the cells seem to retain their activity and PDCA production can likely be extended to reach higher titers. Considering the investment of glucose for biomass formation, using the cells as long and efficient as possible is critical to offset the cost associated with sugar feeding. Despite the high PDCA productivity in bioreactor, the molar conversion yield is reduced compared to shake flask experiments (Section 3.1.1). Likely, the medium impacts the conversion yield as shake flask experiments used phosphate buffer whereas minimal medium was applied in bioreactor experiments. It was demonstrated, that increasing amounts of iron sulfate, which is part of the medium, reduce the molar conversion yield from 68 ± 1% (0.0 g/L iron sulfate heptahydrate) to 57 ± 1% (0.3 g/L iron sulfate heptahydrate) (Supporting Information S1: Figure A.1C). Iron is known to form complexes with PCA that might reduce the substrate availability for LigAB conversion. Here further medium optimization is necessary to overcome these limitations. After production, PDCA can be isolated from fermentation broth for instance by reactive extraction for further processing (Notheisen and Takors 2024).

### 3.2 PDCA Production From Lignin Feedstocks

#### 3.2.1 Evaluation of Lignin Substrates and Pretreatments

In previous work, up to 40 mg/L PDCA were produced from 15 g/L lignin with engineered *P. putida* (Gómez‐Álvarez et al. 2022). Often, studies on lignin degradation use different substrates and pretreatments. Whereas some simply add lignin to water (Spence et al. 2021; Williamson et al. 2020) others performed alkaline pretreatment before bioconversion (Gómez‐Álvarez et al. 2022; Zhao et al. 2021). To identify the optimal pretreatment and lignin substrate for PDCA production, three different lignin substrates and four different pretreatments were screened. Two bacterial strains (*R. jostii* and *P. putida*) are reported to produce PDCA and were included in this experiment. The PDCA‐producing *R. jostii* strain was not available for this study, but its ancestral strain *R. jostii ΔpcaHG* which produces the precursor PCA from lignin conversion (Spence et al. 2021). As *R. jostii ΔpcaHG* is not able to convert PCA into PDCA, *P. putida ligAB* was not induced yielding mainly PCA accumulation as well. Thereby, lignin conversion could be compared via PCA concentration. Nevertheless, minor PDCA formation in the late phase of the experiment was still observed with non‐induced *P. putida ligAB*. To account for the PCA that was converted into PDCA, the lumped amount of PCA and PDCA (P(D)CA) was used for comparison. The lignin substrates included are sodium hydroxide (SH) lignin, Kraft lignin and wheat straw. SH lignin is a commercial soda lignin from wheat straw and was used in previous work on PDCA production from lignin (Gómez‐Álvarez et al. 2022; Spence et al. 2021). Kraft lignin is a different commercially available lignin feedstock and wheat straw was included since it represents a natural lignin substrate.

**Figure 4:**
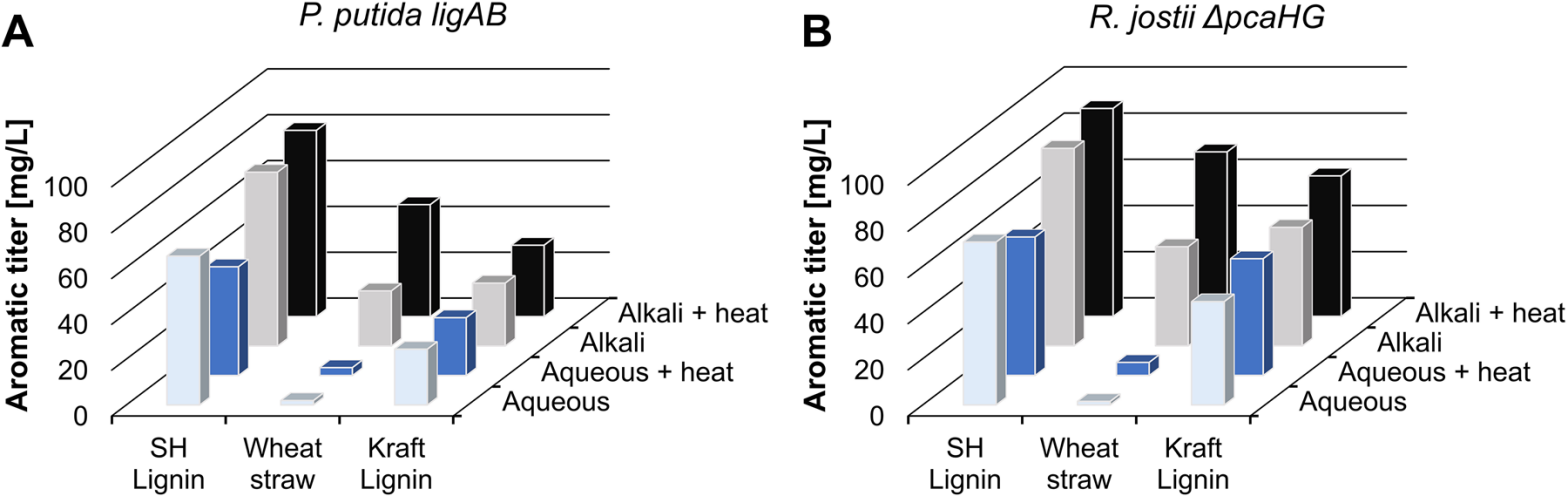
Lignin pretreatment and substrate screening with *P. putida ligAB* (A) and *R. jostii ΔpcaHG* (B). The three lignin substrates sodium hydroxide (SH) lignin, wheat straw and Kraft lignin were pretreated by adding substrate to water (aqueous) or to NaOH in water (pH 13) (alkali). Lignin solutions were used as prepared or autoclaved at 120°C for 20 min (heat). 10 g/L of pretreated lignin substrate in M9 medium were used in the reaction with 4 g/L biomass of either *P. putida ligAB* or *R. jostii ΔpcaHG*. Maximum values for PCA (A) or P(D)CA (B) are presented. The lumped P(D)CA concentration describes the sum of PCA and PDCA. The data represents mean of triplicates. Values and standard deviation can be found in Supporting Information Table A.2.

Maximum (lumped) P(D)CA titers obtained from resting cell conversion of 10 g/L lignin as sole carbon substrate are presented in Figure 4 and kinetics can be found in supplementary Figure A.2 and A.3. The highest PCA titer observed for *R. jostii ΔpcaHG* was 90 ± 11 mg/L for sodium hydroxide (SH) lignin and alkali + heat pretreatment (Supporting Information S1: Table A.1). The same combination also showed highest lumped P(D)CA titer of 81 ± 3 mg/L with *P. putida ligAB*. Therefore, consecutive experiments used SH lignin with alkali + heat pretreatment as substrate. Treating lignin by alkali exposure yielded second highest values whereas dissolving lignin in water provided the lowest (lumped) P(D)CA titers irrespective what aqueous treatment was applied. The trend in PCA or P(D)CA yield for different pretreatments is mirrored in the amount of monoaromatics released by the pretreatment. Vanillic acid and 4‐hydroxybenzoic acid are direct precursors of PCA and are derived from G‐ or H‐lignin unit breakdown. The amount of both is increased after alkali pretreatments compared to aqueous pretreatments (Supporting Information S1: Table A.3).

Various studies outlined the benefit of alkali solubilization of lignin for conversion. Alkali‐solubilised lignin showed improved substrate availability compared to water‐solubilised lignin, for instance investigating *Rhodococcus opacus* (Zhao et al. 2022; Zhao et al. 2021). Several studies further reported that lignin is partly depolymerized when heated at alkaline conditions and monoaromatics like vanillin (Hosoya et al. 2022), p‐coumaric acid (Rodriguez et al. 2017), and guaiacol (Almqvist et al. 2021) were released from the complex lignin matrix. Such depolymerized lignin is a well‐suited substrate for *P. putida* growth and bioconversion (Almqvist et al. 2021; Rodriguez et al. 2017). Considering that 90 mg/L produced PCA corresponds to about 1% converted lignin anticipates that structural decomposition of polymeric lignin may have only occurred in small amounts. Instead, mono‐aromatic building blocks were likely excavated from the matrix serving as substrate for PCA and PDCA production. Nevertheless, coupling bioconversion with alkali depolymerization seems to be promising for increasing the conversion yield.

The different substrates showed significantly different titers for both strains. Highest titers were obtained from SH lignin, followed by Kraft lignin, and wheat straw. Depending on their plant origin, lignin contains varying fractions of H‐, G‐ and S‐monolignol‐derived aromatics. Of those, only H‐ and G‐derived monoaromatics can be converted into PCA. In contrast to the relatively pure lignin substrates SH and Kraft lignin with a lignin content of roughly 90% (Constant et al. 2016), the lignin fraction of wheat straw accounts for only 11%–26% (Zhang et al. 2022). Considering the lignin fraction in the substrate, an efficient conversion of the lignin can be noted for the wheat straw substrate. Of note, carbohydrates released from wheat straw are not expected to significantly contribute to P(D)CA formation due to a shortage of PCA synthetase (Li and Ye 2021). Li and Ye 2021 demonstrated that only 5 mg/L PCA were produced from 40 g/L glucose within 48 h of fermentation with a *P. putida* strain that bears a *pcaHG* knockout.

Most likely, the aromatics released into solution by pretreatment were used for PCA formation (Supporting Information S1: Figure A.4 B). Sodium hydroxide and Kraft lignin differ in structure, molecular weight, and aromatic composition. Comparing both, SH lignin contains a higher percentage of mono‐aromatics and has a lower molecular weight compared to Kraft lignin (Constant et al. 2016). This might explain the highest (lumped) P(D)CA yields for SH lignin hypothesizing that product formation predominantly occurred with the released aromatic compounds. The literature values for microbially produced monoaromatics as end product of lignin conversion are in the range of the produced PCA and P(D)CA in this study. Williamson et al. 2020 observed production of 89 mg/L ferulic acid and coumaric acid from conversion of 20 g/L aqueous + heat pretreated SH lignin with engineered *P. putida*. Sainsbury et al. 2013 accumulated 96 mg/L vanillin, ferulic acid, and 4‐hydroxybenzaldehyde from conversion of 25 g/L aqueous + heat pretreated wheat straw lignin with engineered *R. jostii*.

Interestingly, PCA degradation was observed for some *R. jostii ΔpcaHG* cultivations in this study (Supporting Information S1: Figure A.3). The β‐ketoadipate pathway is one major pathway for PCA degradation and was eliminated by the knock‐out of *pcaHG* which encodes the protocatechuate‐3,4‐dioxygenase, the first enzyme in this pathway (Spence et al. 2021). However, Spence et al. 2020 discovered an alternative PCA pathway that allowed PCA metabolization via hydroxyquinol in *R. jostii ΔpcaHG*. Eliminating this competing pathway could prevent the non‐wanted consumption of PCA.

#### 3.2.2 PDCA Production From SH Lignin

**Figure 5:**
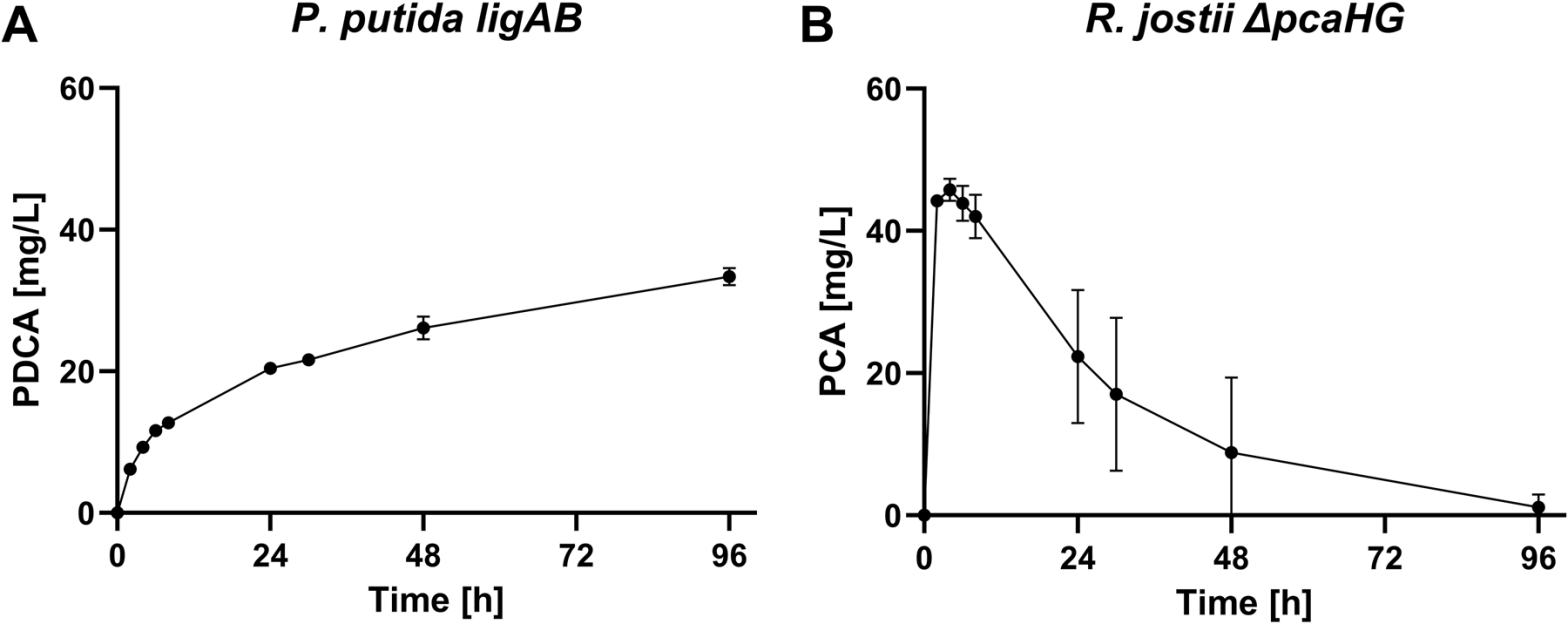
PDCA or PCA production from lignin with *P. putida ligAB* (A) or *R. jostii ΔpcaHG* (B) in bioreactor. 5.7 g/L SH lignin (alkali + heat pretreated) were added to 13 g/L *P. putida ligAB* or 17 g/L *R. jostii ΔpcaHG* resting cells after the initial growth phase. The data represents mean of duplicates with variation (error bars).

The PDCA and PCA production from SH lignin was transferred into a bioreactor scale using *P. putida ligAB* or *R. jostii ΔpcaHG*. The same protocol was applied as for PCA conversion described in Section 3.1.2. First, biomass was produced using glucose as carbon source and, after complete glucose depletion, lignin was added to those resting cells. During the 96 h of incubating *P. putida ligAB* with 5.7 g/L SH lignin, 33 ± 1 mg/L PDCA accumulated between the process time 2–8 h with the biomass specific productivity of 0.1 ± 0.0 mg/g/h (Figure 5A) while PCA did not occur. On contrary, PCA titers rose rapidly with *R. jostii ΔpcaHG* at a biomass‐specific production rate of 1.3 ± 0.0 mg/g/h (0–2 h) showing the maximum of 46 ± 2 mg/L after 4 h (Figure 5B). Then, PCA degraded as discussed in Chapter 3.2.1.

### 3.3 Mixed Culture Improves Lignin Conversion and PDCA Production

PDCA production from lignin with *P. putida ligAB* suffers from low biomass specific productivity (0.1 mg/g/h, Section 3.2.2). In contrast, PDCA production from PCA is about 100‐fold faster (biomass specific productivity of 14 ± 1 mg/g/h, Section 3.1.1). Apparently, access to the lignin‐derived precursors finally limits the conversion capacities of *P. putida ligAB*. To overcome these limitations, we approached a strategy based on the development of a synthetic microbial mixed culture between *P. putida ligAB* and the lignin‐degrading bacterium *R. jostii ΔpcaHG*. As demonstrated in Section 3.2.1, *R. jostii ΔpcaHG* accumulates PCA with a biomass‐specific productivity of up to 2.3 ± 0.6 mg/g/h (0‐6 h). It was assumed that the produced PCA from *R. jostii ΔpcaHG* can serve as substrate for accelerated PDCA production with *P. putida ligAB*. Resting cell shake flask experiments were performed to evaluate the effect of a mixed culture with a total biomass concentration of 15–16 g/L in the reaction. The experiment was performed with 3 or 6 g/L *P. putida ligAB* with 13 g/L *R. jostii ΔpcaHG or 9 g/L R. jostii ΔpcaHG*. For comparison, 3 or 6 g/L *P. putida ligAB* were cultivated in single culture without added *R. jostii ΔpcaHG*. Since the cells are not growing, the strain ratio was expected to be constant during the time of the experiment.

**Figure 6:**
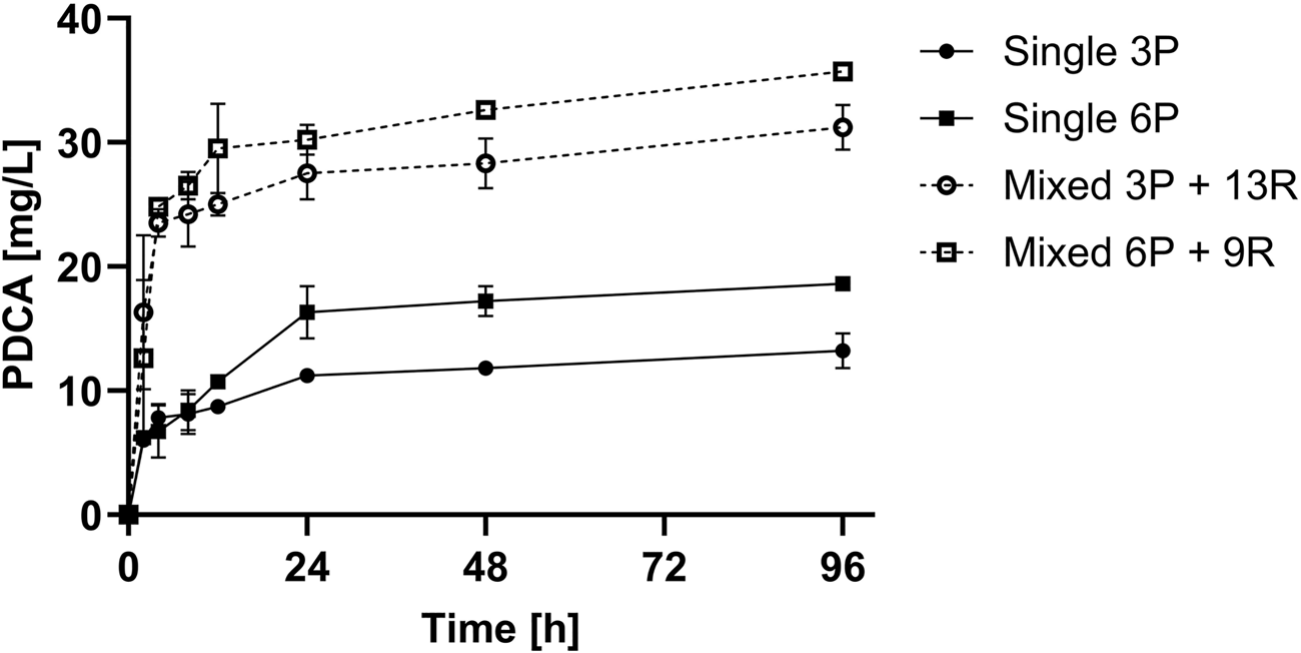
PDCA production from lignin with microbial mixed culture of *P. putida ligAB* and *R. jostii ΔpcaHG*. 6.7 g/L SH lignin (alkali + heat pretreated) were added to different biomass ratios of resting cells. For single culture (single), lignin was incubated with 3 or 6 g/L *P. putida ligAB* (3 P or 6 P, solid lines). For mixed culture (mixed), 13 or 9 g/L *R. jostii ΔpcaHG* were added to 3 or 6 g/L *P. putida ligAB* biomass (3 P + 13 R or 6 P + 9 R, dashed lines) to reach a total biomass concentration of 15‐16 g/L. The data represents the mean of duplicates with variation (error bars).

The addition of *R. jostii ΔpcaHG* to *P. putida ligAB* biomass for PDCA production from lignin increased the PDCA production in two ways (Figure 6). First, *P. putida ligAB* biomass‐specific PDCA productivities were almost doubled comparing single and mixed culture (Table 1). Second, the total amount of PDCA produced from lignin after 96 h was doubled. The biomass‐specific PDCA productivity increased from 0.6 ± 0.1 mg/g/h to 1.9 ± 0.1 mg/g/h for 3 g/L *P. putida ligAB* in single and mixed culture. The addition of *R. jostii ΔpcaHG* with biomass‐specific PCA productivity of 2.3 mg/g/h could help increase the PDCA productivity of *P. putida ligAB*. Interestingly enough, the achieved PDCA productivity of 1.9 mg/g/h in mixed culture corresponds to the PCA productivity of *R. jostii ΔpcaHG*. A detailed comparison of productivity for the individual strains or mixed cultures at equal biomass can be found in Supporting Information S1: Table A.4.

**Table 1:** PDCA productivities from lignin with mixed culture. 6.7 g/L SH lignin (alkali + heat pretreated) were added to resting cells of 3 or 6 g/L *P. putida ligAB* (single 3 P or single 6 P) or 3 or 6 g/L *P. putida ligAB* with 13 or 9 g/L *R. jostii ΔpcaHG* (mixed 3 P + 13 R or mixed 6 P + 9 R). Volumetric and biomass‐specific (*P. putida ligAB)* productivities were calculated for the first 4 h of reaction.

	Single 3 P	Single 6 P	Mixed 3 P + 13 R	Mixed 6 P + 9 R
*P. putida ligAB [g/L]*	3	6	3	6
*R. jostii ΔpcaHG [g/L]*	‐‐‐	‐‐‐	13	9
Volumetric productivity [mg/L/h]	1.9 ± 0.3	1.7 ± 0.5	5.9 ± 0.3	6.2 ± 0.0
Specific productivity [mg/g_*P. putida*_/h	0.6 ± 0.1	0.3 ± 0.1	1.9 ± 0.1	1.0 ± 0.0

*Note:* The data represents mean of duplicates with variation.

Saturation of PDCA production was reached after 96 h at 19 ± 0 mg/L PDCA with 6 g/L *P. putida ligAB* and 36 ± 0 mg/L PDCA with 6 g/L *P. putida ligAB* + 9 g/L *R. jostii ΔpcaHG*.

The enhanced PDCA formation observed in mixed culture can be attributed to an additive effect of *R. jostii ΔpcaHG*, as the improved performance is directly linked to increased substrate availability provided by this strain. Whereas no lignin depolymerization activity has been reported for *P. putida*, strains of *R. jostii* presumably are able to depolymerize lignin to some extent according to the literature (Ahmad et al. 2010), thereby increasing the fraction of available monoaromatics. This could explain that the combination of both strains leads to increased substrate usage and higher PDCA yields. However, the exact mechanisms underlying *R. jostii* lignin depolymerization remain unclear.

The data indicate that PDCA production from lignin is limited by the supply with PCA from lignin conversion. This limitation was partly overcome by the addition of *R. jostii ΔpcaHG*. Thereby, the strain ratio had only negligible effects—most likely because the maximum lignin degradation capacity of *R. jostii ΔpcaHG* was already reached. It was tested that amounts above 5 g/L of *R. jostii ΔpcaHG* cells in single culture did not further improve lignin degradation (Supporting Information S1: Figure A.5).

Other literature also reports the advantages of mixed culture. Cai et al. 2022 observed 10% enhanced lignin conversion for a mixed culture of *Sphingobium* and *Rhodococcus opacus*. Thereby, 87 mg/L muconic acid was produced from 4 g/L lignin.

In this study, the benefit of a synthetic microbial mixed culture for PDCA production from lignin was demonstrated. The strain *R. jostii ΔpcaHG* facilitates lignin degradation and helps providing precursors to *P. putida ligAB* for PDCA production—mimicking natural lignin degradation. To achieve complete microbial lignin degradation, a fine‐tuned microbial consortium might be necessary. White‐rot fungi are the main organisms involved in lignin depolymerization, whereas bacteria are often specialized for low‐molecular‐weight lignin degradation (Kamimura et al. 2019). Including white‐rot fungi in a mixed culture can lead to enhanced lignin conversion as tested by Salvachúa et al. 2016. In their work, the secretome of fungi was used to depolymerize lignin and funnel the lignin‐released monoaromatics as substrate for *P. putida*.

As well, the introduction of heterologous genes involved in lignin depolymerization like the lignin‐oxidizing enzyme Dyp2 into bacterial hosts might help increasing the lignin conversion (Spence et al. 2021). Additionally, the expression of further auxiliary enzymes might be necessary to prevent lignin repolymerization as outlined in Liang et al. 2025.

## 4 Conclusions

Continuous PDCA production with *P. putida ligAB* was performed in 30 L scale with 1.9 g/L constant PDCA production from PCA. Studies identified the duration of induction of *ligAB* as a sensitive parameter to overcome limitations of PCA to PDCA conversion which peaked with 77% yield. Using lignin, both, substrate source and its preparation have a significant impact on the obtained PCA yield. Despite optimized substrate feeding, PDCA production suffered from the too slow supply of precursors by *P. putida ligAB*. This was solved by adding the lignin‐degrading bacterium *R. jostii ΔpcaHG* that managed to double the productivity from lignin conversion. Accordingly, the study outlines the sensitive steps of PDCA production from lignin that need to be considered for scaling up. Considering the proper induction of *ligAB* as a prerequisite, the consideration of a “helper” strain that improves lignin degradation may motivate tests in other applications. Furthermore, the current study outlines the potential of resting cells for production which may motivate future developments. They enabled continuous operation that finally opens the door for maximized volumetric productivities, a prerequisite for competitive bioprocesses.

## Author Contributions

Jan Seeger and Ralf Takors conceptualized the study. Jan Notheisen and Susanne Müller performed the experiments. Jan Notheisen analyzed the experimental data. Helena Gómez‐Álvarez, Eduardo Díaz, Goran M.M. Rashid, and Timothy D.H. Bugg provided the engineered strains for this study. Eduardo Díaz, Timothy D.H. Bugg, and Ralf Takors acquired the funding for this study. The work was supervised by Ralf Takors. Jan Notheisen prepared the original draft and all authors then reviewed and edited the manuscript. All authors approved the manuscript.

## Conflicts of Interest

The authors declare no conflicts of interest.

## Supporting information

Appendix A revised.

## Data Availability

All data generated or analyzed during this study are included in this article and its additional file. Raw data are publicly available in the DaRUS repository (https://doi.org/10.18419/DARUS-4525). Please contact the corresponding author for further information.
