# Supplementary material for: Scaling‐up the Bioconversion of Lignin to 2,4‐Pyridinedicarboxylic Acid With Engineered Pseudomonas putida for Bio‐Based Plastics Production
Source: Biotechnol Bioeng. 2025 Jul 14;122(10):2770–80. doi: 10.1002/bit.70020 (PMC12417764; doi:10.1002/bit.70020)
Supplement: Supplementary file 1 — Appendix A revised. [file BIT-122-2770-s001.pdf]

Appendix A Supplementary data

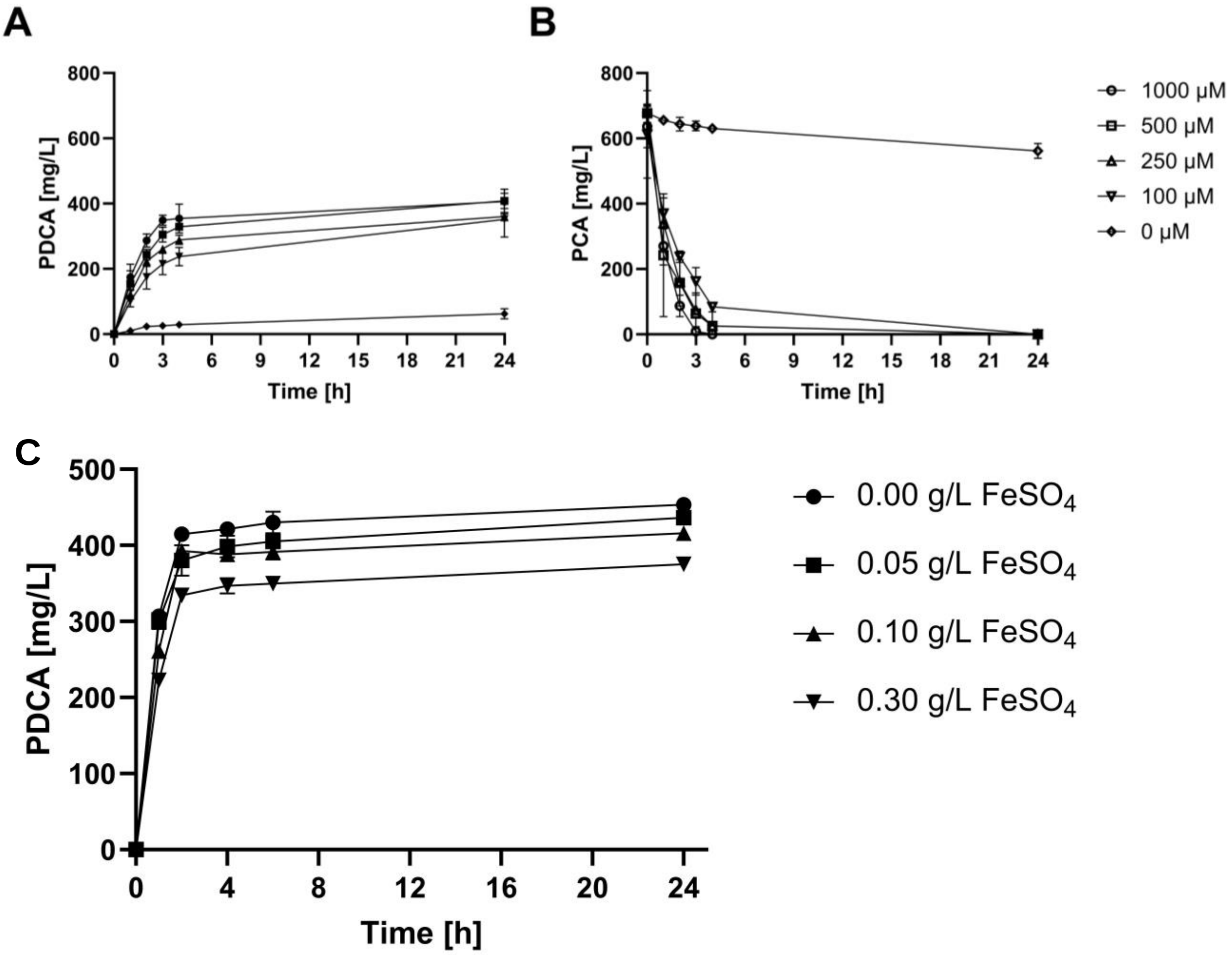

Figure A.1 PDCA production from PCA. Effect of inducer concentration in pre-culture on PDCA production (**A** & **B**). PDCA (**A**) was produced with 10 g/L resting cells of *P. putida ligAB* from PCA (**B**) in phosphate buffer. The cells were incubated for 6 h with different concentrations of IPTG from 0 to 1000 µM during pre-culture. The data represents the mean and standard deviation (error bars) of triplicates. **C** Effect of iron sulfate on PDCA production. Different amounts of iron sulphate (heptahydrate) from 0.0 to 0.3 g/L were added to 20 g/L *P. putida ligAB* (obtained from bioreactor cultivation) in phosphate buffer. The data represents mean and variation of duplicates (error bars).

Table A.1 Substrate and pre-treatment screening. Lignin pre-treatment and substrate screening with *P. putida ligAB* or *R. jostii ΔpcaHG*. Sodium hydroxide (SH) lignin, wheat straw and Kraft lignin were pre-treated by aqueous or alkali (pH13) pre-treatment with and without autoclavation (heat). 10 g/L lignin substrate were incubated with 4 g/L biomass. The cells were obtained from preculture in M9 medium with 5 g/L glucose. Thereby, the expression of *ligAB* in *P. putida ligAB* was left uninduced which led to the accumulation of PCA predominatly to allow better comparison with *R. jostii ΔpcaHG.* Still, small amounts of PDCA accumulated and where condiered when calculating the lumped amount of monoaromatics (P(D)CA = PDCA + PCA). Cells were harvested by centrifugation and resuspended in lignin medium for conversion. The approach was incubated for 5-7 days at 30 °C, shaking. Results from two-way ANOVA and post-hoc test comparison of data from maximum titers with errors were used for ANOVA. Group means are presented for both strains. Significant different groups (α = 5 %) were indicated by a small letter (a-c).

R. jostii ΔpcaHG		**PCA [mg/L]**	**Group**	***P. putida ligAB***		**P(D)CA [mg/L]**	**Group**
Substrate	SH lignin	76.5 ± 13.6	a	Substrate	SH lignin	67.3 ± 14.1	a
	Kraft lignin	51.8 ± 6.1	b		Kraft lignin	27.0 ± 4.9	b
	Wheat straw	30.3 ± 29.9	c		Wheat straw	19.5 ± 19.9	c
Treatment	Aqueous	38.9 ± 30.2	c	Treatment	Aqueous	30.4 ± 28.1	c
	Aqueous+heat	38.7 ± 25.2	c		Aqueous+heat	25.3 ± 19.1	c
	Alkali	59.9 ± 19.8	b		Alkali	42.4 ± 25.1	b
	Alkali + heat	74.0 ± 14.0	a		Alkali + heat	53.7 ± 22.5	a

Table A.2 Substrate and pre-treatment screening values from figure 4. Lignin pre-treatment and substrate screening with *P. putida ligAB* or *R. jostii ΔpcaHG*. Sodium hydroxide (SH) lignin, wheat straw and Kraft lignin were pre-treated by aqueous or alkali (pH13) pre-treatment with and without autoclavation (heat). 10 g/L lignin substrate were incubated with 4 g/L biomass. The cells were obtained from preculture in M9 medium with 5 g/L glucose. Thereby, the expression of *ligAB* in *P. putida ligAB* was left uninduced which led to the accumulation of PCA predominantly to allow better comparison with *R. jostii ΔpcaHG.* Still, small amounts of PDCA accumulated and where considered when calculating the lumped amount of monoaromatics (P(D)CA = PDCA + PCA). Cells were harvested by centrifugation and resuspended in lignin medium for conversion. The approach was incubated for 5-7 days at 30 °C, shaking. Mean and standard deviation (error) of triplicates are shown.

P.putida ligAB				
	Aqueous	Aqueous+heat	Alkali	Alkali+heat
SH lignin	65 ± 9 mg/L	47 ± 1 mg/L	76 ± 2 mg/L	81 ± 3 mg/L
Wheat straw	2 ± 1 mg/L	3 ± 1 mg/L	24 ± 2 mg/L	49 ± 1 mg/L
Kraft lignin	24 ± 22 mg/L	25 ± 1 mg/L	27 ± 1 mg/L	31 ± 9 mg/L
R. jostii ΔpcaHG				
SH lignin	70 ± 4 mg/L	60 ± 2 mg/L	86 ± 5 mg/L	90 ± 11 mg/L
Wheat straw	2 ± 1 mg/L	6 ± 1 mg/L	43 ± 3 mg/L	71 ± 4 mg/L
Kraft lignin	45 ± 1 mg/L	51 ± 1 mg/L	51 ± 1 mg/L	61 ± 1 mg/L

Table A.3: Content of 4-hydroxybenzoic acid (4-HBA) and vanillic acid (VA) in 10 g/L lignin after different pre-treatments. Three different lignin substrates were analyzed: industrial sodium hydroxide (SH) lignin, wheat straw (chopped to 1 cm pieces), and commercial Kraft lignin.

		**4-HBA [mg/L]**	**VA [mg/L]**
SH lignin	Aqueous	5.7 ± 1.3	10.7 ± 3.3
	Aqueous+heat	4.5 ± 0.7	7.8 ± 1.2
	Alkali	5.8 ± 0.6	22.8 ± 3.4
	Alkali+heat	6.3 ± 1.0	26.3 ± 5.9
Wheat straw	Aqueous	0.2 ± 0.1	0.3 ± 0.2
	Aqueous+heat	0.2 ± 0.2	0.3 ± 0.2
	Alkali	0.5 ± 0.0	1.0 ± 0.2
	Alkali+heat	1.1 ± 0.2	3.0 ± 0.3
Kraft lignin	Aqueous	0.8 ± 0.2	9.4 ± 0.6
	Aqueous+heat	0.6 ± 0.6	12.7 ± 1.3
	Alkali	1.2 ± 0.1	21.2 ± 3.1
	Alkali+heat	1.1 ± 0.9	29.0 ± 2.8

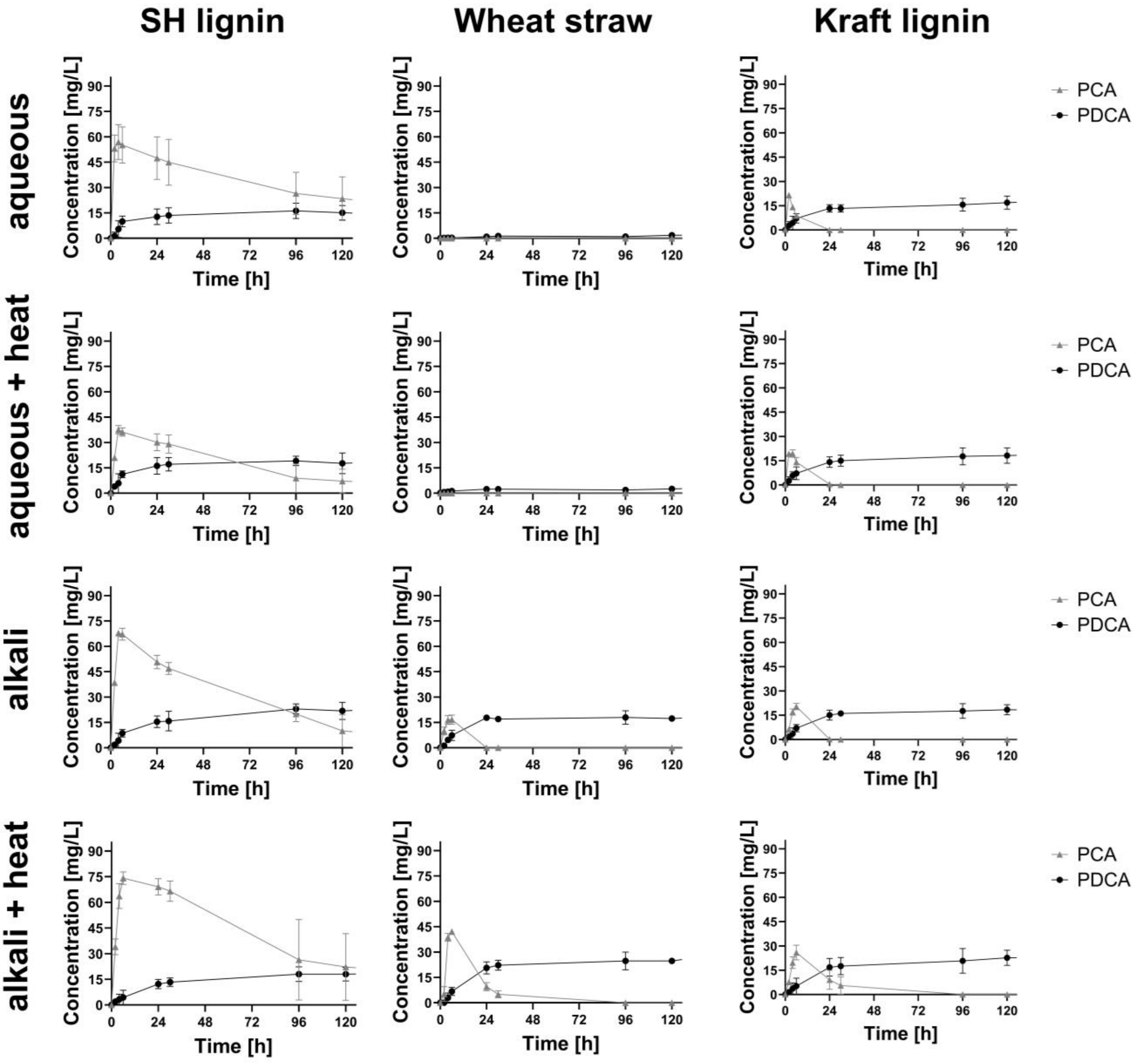

Figure A.2 Lignin pre-treatment and substrate screening with *P. putida ligAB.* Sodium hydroxide (SH) lignin, wheat straw and Kraft lignin were pre-treated by dissolving in water (aqueous) or dissolving in NaOH at pH 13 (alkali). Lignin solutions were used as prepared or autoclaved at 120 °C for 20 min (heat). 10 g/L lignin substrate as sole carbon source in M9 medium were used in the reaction with 4 g/L biomass. The cells were obtained from preculture in M9 medium with 5 g/L glucose. Thereby, the induction of *ligAB* expression was left uninduced to accumulate majorily PCA for comparison with *R. jostii ΔpcaHG.* Cells were harvested by centrifugation and resuspended in lignin medium for conversion. The approach was incubated for 7 days at 30 °C, shaking. The data represents the mean and standard deviation (error bars) of triplicates.

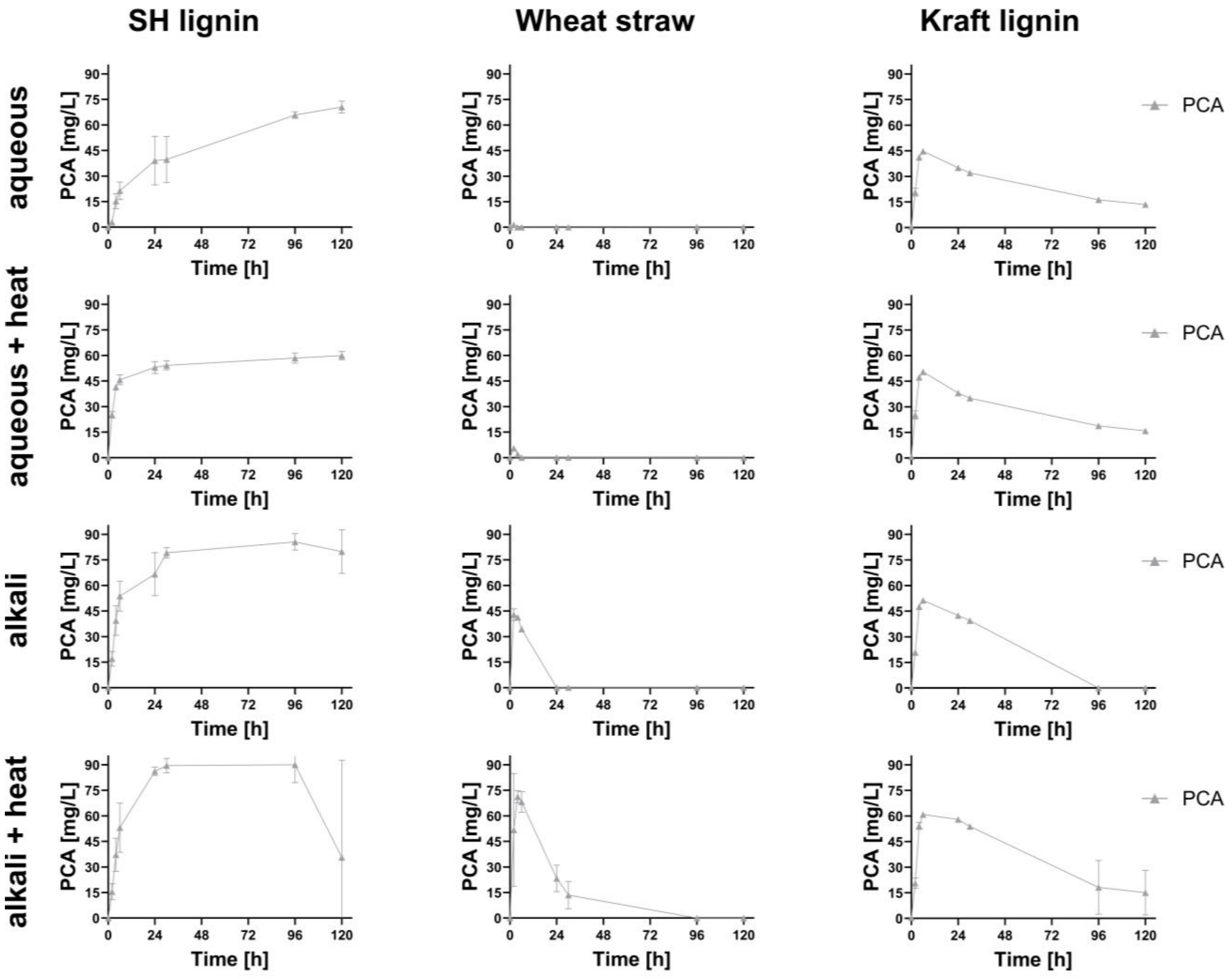

Figure A.3 Lignin pre-treatment and substrate screening with *R. jostii ΔpcaHG.* Sodium hydroxide (SH) lignin, wheat straw and Kraft lignin were pre-treated by dissolving in water (aqueous) or dissolving in NaOH at pH 13 (alkali). Lignin solutions were used as prepared or autoclaved at 120 °C for 20 min (heat). 10 g/L lignin substrate in M9 medium were used in the reaction with 4 g/L biomass. The cells were obtained from preculture in M9 medium with 5 g/L glucose. Cells were harvested by centrifugation and resuspended in lignin medium for conversion. PCA concentrations were monitored The approach was incubated for 5 days at 30 °C, shaking. The data represents the mean and standard deviation (error bars) of triplicates.

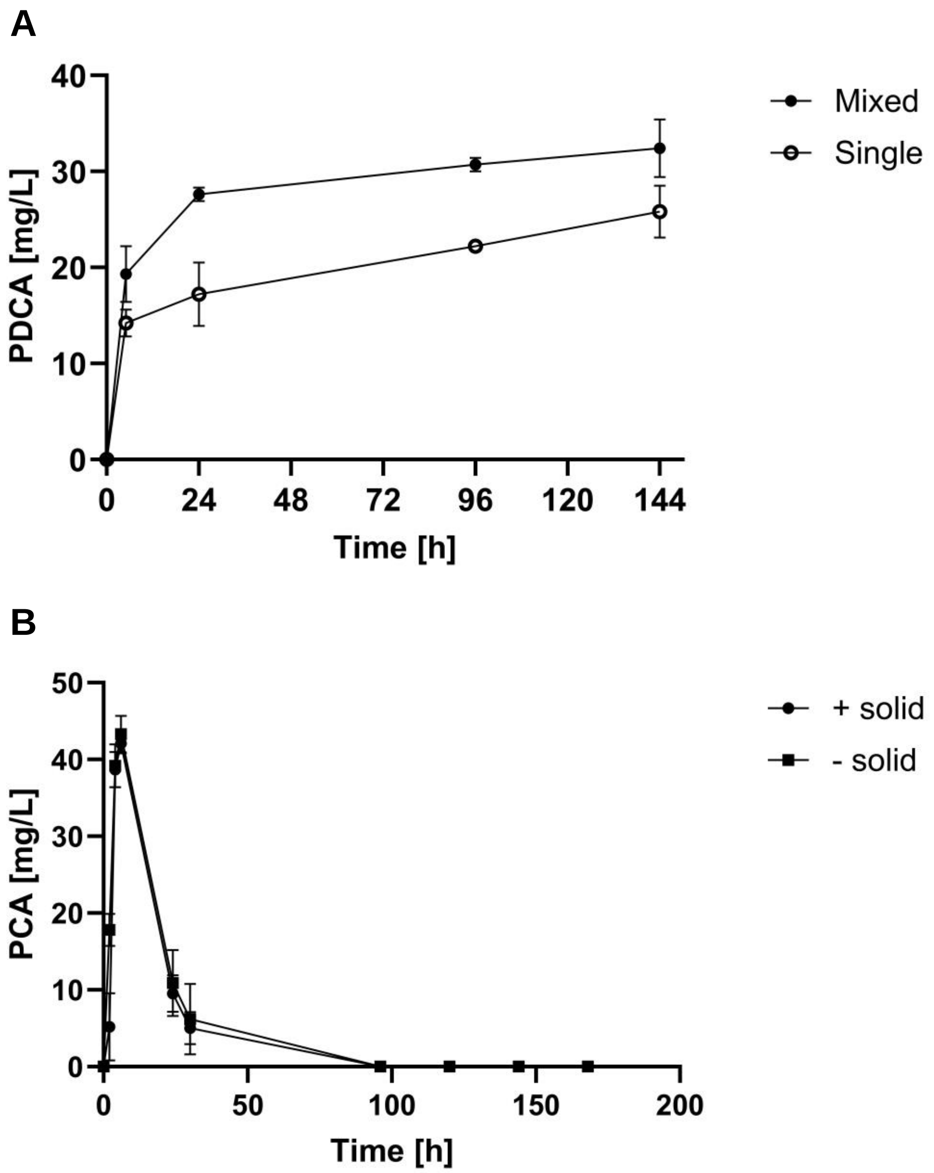

Figure A.4 Microbial lignin conversion. A Comparison of mixed and single culture at equal biomass levels. 10 g/L SH lignin (alkali + heat pre-treated) were added to resting cells of 4.5 g/L *P. putida ligAB* (Single) or 1.5 g/L *P. putida ligAB* with 3 g/L *R. jostii ΔpcaHG* (Mixed). Cells were obtained from shaking flask pre-culture. The data represents mean of duplicates with variation (error bars). **B** Comparison of 10 g/L wheat straw conversion with 4 g/L *P. putida ligAB* (uninduced) with and without solid wheat straw particles in the reaction. A 20 g/L wheat straw stock solution was prepared from alkali + heat pre-treatment. The stock was used with undissolved solids (+solid) or centrifuged at 5,750 rcf for 10 min before adding the supernatant to the reaction (-solid). The data represents mean and standard deviation (error bars) of triplicates.

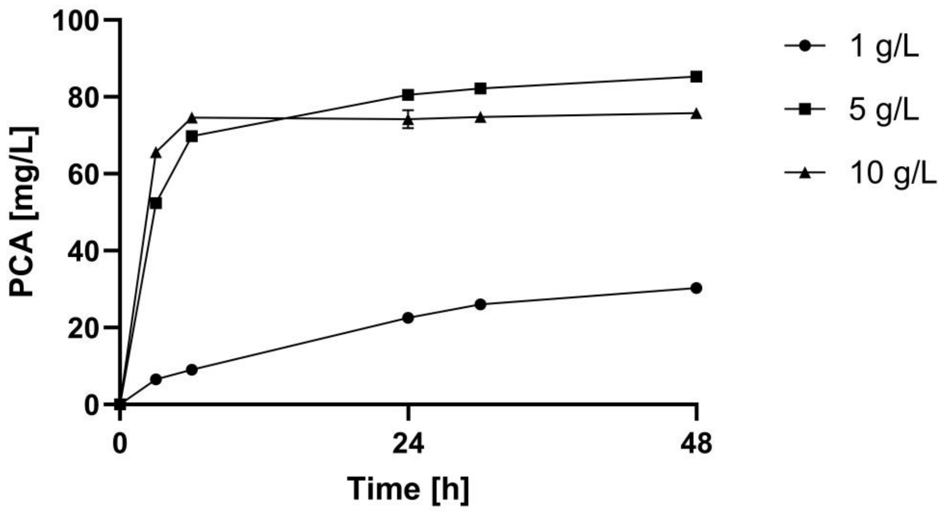

Figure A.5 Lignin degradation with different concentrations of *R. jostii ΔpcaHG* cells. Resting cell cultures of R. jostii ΔpcaHG were incubated with 10 g/L alkali+heat pre-treated SH lignin in M9 minimal medium. Cells were obtained from shake flask pre-cultures, harvested by centrifugation, and used at 1, 5, and 10 g/L final biomass concentrations. The data represents mean of duplicates with standard error (error bars).

Table A.4: Comparison of lignin degradation for single or mixed cultures of *P. putida ligAB* and *R. jostii ΔpcaHG*. 10 g/L alkali+heat pre-treated SH lignin was incubated with 2.25 g/L *P. putida ligAB* (Single 2.25P), 2.25 g/L *R. jostii ΔpcaHG* (Single 2.25R), or 2.25 g/L *P. putida ligAB* mixed with 2.25 g/L *R. jostii ΔpcaHG* (Mixed 2.25P+2.25R). The *P. putida ligAB* specific PDCA productivity or *R. jostii ΔpcaHG* specific PCA productivity within the first 6 hours of the reaction is shown, as well as the PDCA or PCA titer after 144 h of incubation at 30 °C on a rotary shaker at 120 rpm. The data represents mean and error from duplicates.

	Single 2.25P	Single 2.25R	Mixed 2.25P + 2.25R
Specific productivity [mg/g/h]	0.7 ± 0.0 PDCA	2.0 ± 0.2 PCA	1.7 ± 0.1 PDCA
Titer 144 h [mg/L]	15.7 ± 1.0 PDCA	56.6 ± 3.2 PCA	37.9 ± 0.6 PDCA
